# Placental Ischemia Says “NO” to Proper NOS-Mediated Control of Vascular Tone and Blood Pressure in Preeclampsia

**DOI:** 10.3390/ijms222011261

**Published:** 2021-10-19

**Authors:** Ana C. Palei, Joey P. Granger, Frank T. Spradley

**Affiliations:** 1 Department of Surgery, University of Mississippi Medical Center, Jackson, MS 39216, USA; apalei@umc.edu; 2 Department of Physiology & Biophysics, University of Mississippi Medical Center, Jackson, MS 39216, USA; jgranger@umc.edu; 3 Department of Pharmacology & Toxicology, University of Mississippi Medical Center, Jackson, MS 39216, USA

**Keywords:** intrauterine growth restriction, nitric oxide, nitric oxide synthases, potential therapies, preeclampsia, pregnancy

## Abstract

In this review, we first provide a brief overview of the nitric oxide synthase (NOS) isoforms and biochemistry. This is followed by describing what is known about NOS-mediated blood pressure control during normal pregnancy. Circulating nitric oxide (NO) bioavailability has been assessed by measuring its metabolites, nitrite (NO_2_) and/or nitrate (NO_3_), and shown to rise throughout normal pregnancy in humans and rats and decline postpartum. In contrast, placental malperfusion/ischemia leads to systemic reductions in NO bioavailability leading to maternal endothelial and vascular dysfunction with subsequent development of hypertension in PE. We end this article by describing emergent risk factors for placental malperfusion and ischemic disease and discussing strategies to target the NOS system therapeutically to increase NO bioavailability in preeclamptic patients. Throughout this discussion, we highlight the critical importance that experimental animal studies have played in our current understanding of NOS biology in normal pregnancy and their use in finding novel ways to preserve this signaling pathway to prevent the development, treat symptoms, or reduce the severity of PE.

## 1. Introduction

The number of hypertensive pregnancies has been on the rise over the past several decades. Hypertension is a leading complication during pregnancy in the United States and throughout the world. One particularly dangerous form is preeclampsia (PE), which in addition to new-onset hypertension, is diagnosed alongside other co-morbidities including proteinuria, oliguria, pulmonary edema, epigastric pain, impaired liver function, thrombocytopenia, headaches, oligohydramnios, placental abruption, and/or fetal growth restriction occurring during the latter half of pregnancy [1]. PE can be classified into two subtypes of early- or late-onset, with a greater number of diagnostic co-morbidities being an index of the severity of PE. More severe forms of PE are associated with maternal blood pressure reaching >160/110 mmHg, and the rate of PE with severe features is rising [2]. PE not only has immediate outcomes leading to maternal and/or fetal morbidity and mortality, but also has long-term ramifications with increased risk for future cardiovascular disease in formerly-preeclamptic women and their offspring [3,4]. Overall, the diverse features and continued immediate and long-term impacts of PE highlight our lack of a full appreciation of the organ systems and mechanisms involved in the development of hypertension in PE.

A pregnancy-specific organ implicated in the progression of PE is the diseased placenta. The placenta is highly-vascularized and is the site where nutrient and waste exchange occur to ensure proper fetal growth. Placentation involves invasion of fetal-derived trophoblast cells that promote decidualization of the uterus with remodeling and widening of the maternal spiral arteries, as reviewed in [5]. Damage to the fetal-derived trophoblast cells that reside in the placenta and decidualized uterus is thought to mediate and propagate the maternal vascular dysfunction and hypertensive outcomes in PE, regardless of its gestational age of presentation [6,7]. Such damage can result from multiple factors, including malperfusion and resultant placental ischemia. A method by which this is measured is Doppler ultrasound with the observation of dicrotic notches in the pulse wave signal of the uterine artery during PE [8]. This notching is indicative of an increased uterine vascular resistance index (UARI) [9]. Uteroplacental ischemia is detected in PE, especially with more severe forms [10]. Placental ischemia/hypoxia elicits the release of factors that target the endothelium and reduce the ability of maternal nitric oxide synthase (NOS) to modulate vascular tone and blood pressure.

In this review, we first provide a brief overview of the NOS isoforms and biochemistry of these enzymes. This is followed by describing what is known about NOS-mediated blood pressure control during normal pregnancy. Indeed, circulating nitric oxide (NO) bioavailability has been assessed by measuring its metabolites, nitrite (NO_2_), and/or nitrate (NO_3_), and shown to rise throughout normal pregnancy in humans and rats [11,12,13,14] and decline postpartum [15,16]. In contrast, placental ischemia leads to systemic reductions in NO bioavailability leading to maternal endothelial and vascular dysfunction with subsequent development of hypertension in PE [17]. We end this article by describing emergent risk factors for placental malperfusion and ischemic disease and discuss strategies to therapeutically target the NOS system to increase NO bioavailability in preeclamptic patients. Throughout this discussion, we highlight the critical importance that experimental animal studies have played in our current understanding of NOS biology in normal pregnancy and their use in finding novel ways to preserve this signaling pathway to prevent the development, treat symptoms, or reduce the severity of PE.

## 2. NOS Isoforms and Biochemistry

NO is a ubiquitous gaseous and lipophilic molecule involved in a variety of biological processes. NO is generated from the conversion of L-arginine to L-citrulline. In mammals, this reaction is catalyzed by three isoforms of the enzyme nitric oxide synthase: neuronal (NOS1), inducible (NOS2), and endothelial (NOS3). An overview of the NOS structure, regulation, and function have been reviewed in depth elsewhere [18,19,20]. But briefly, each NOS enzyme is encoded by a different gene, with 51–57% homology between the isoforms. They have different cell localization, regulation, catalytic properties, and inhibitor sensitivity. NOS1 and NOS3 are constitutively expressed, usually producing low concentrations of NO for paracrine signaling related mainly to neurotransmission and cardiovascular homeostasis (control of vascular tone, cellular proliferation, leukocyte adhesion, and platelet aggregation). Whereas, NOS2 expression is induced by cytokines and typically generates high concentrations of NO for modulation of inflammatory responses, host defense against pathogens, and airway epithelial formation. Yet, numerous other stimuli may regulate NOS at the transcriptional, posttranscriptional, and posttranslational levels. In this regard, variations in the nucleotide sequence of the NOS genes have been reported to alter NOS synthesis and activity. Consequently, these genetic polymorphisms may affect NO production. For instance, haplotypes formed by the combination of the NOS3 polymorphisms T-786C in the promoter region, G894T in exon 7 (Glu298Asp), and a 27 bp variable number of tandem repeats (VNTRs) a/b in intron 4 have been associated with susceptibility to the development of disease, decreased circulating NO levels, and lack of response to antihypertensive treatment in PE [21].

In its active form, NOS is a homodimer where each subunit is composed of a C-terminal reductase domain, which comprises the binding sites for nicotinamide adenine dinucleotide phosphate (NADPH), flavin mononucleotide (FMN), and flavin adenine dinucleotide (FAD), and by an N-terminal oxygenase domain, which contains binding sites for heme, zinc, tetrahydrobiopterin (BH4), and L-arginine. Between the reductase and oxygenase domains, there is a calmodulin-binding sequence. While the Ca^2+^-calmodulin complex is necessary to activate NOS1 and NOS3, NOS2 is already bound to calmodulin and does not depend on Ca^2+^ to be fully active. In the final steps of NO formation, NOS flavins transfer electrons from NADPH to the heme; molecular oxygen binds to heme, and is then incorporated into L-arginine to form NO and L-citrulline. Binding of NOS substrates and cofactors must be finely controlled in order for NO to be efficiently produced. Disruption of this highly coordinated reaction impairs NOS activity. Indeed, limited quantities of substrate and cofactors or excess amounts of endogenous inhibitors, such as asymmetric dimethyl-L-arginine (ADMA) and monomethyl-L-arginine (L-NMMA), may lead to a shift from the dimeric to monomeric form of the enzyme. When uncoupled, NOS generates superoxide anion instead of NO. Furthermore, the interaction of NO with superoxide anion yields peroxynitrite and this highly toxic compound reacts with DNA, proteins, and lipids to cause oxidative stress. On the other hand, oxidative stress enhances the expression of arginase, an enzyme that degrades L-arginine into ornithine and urea. In PE, there is evidence of increased arginase activity, elevated ADMA and superoxide levels, and post-translational modifications of NOS3 by lipid peroxidation aldehydes, all resulting in impaired NOS activity and reduced NO bioavailability [22].

**Figure 1:**
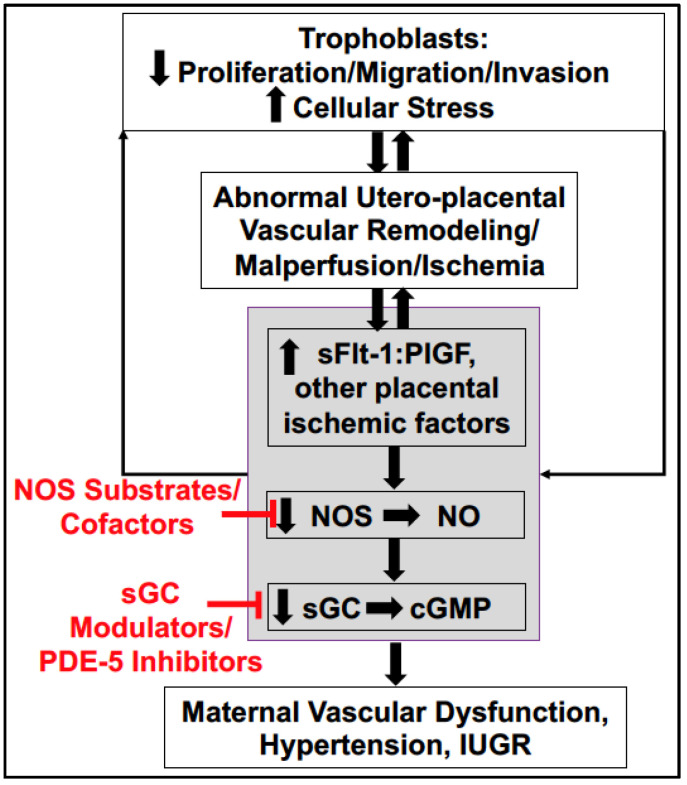
Schematic representation of the cascade of events leading from trophoblast dysfunction and cellular stress to subsequent abnormalities in uteroplacental vascular remodeling, malperfusion, and ischemia. These ischemia/hypoxic events elicit the release of anti-angiogenic and pro-hypertensive factors, like soluble Fms-like tyrosine kinase (sFlt-1), into the maternal circulation. This factor can feedback to reduce cellularity of the placenta, and reduce uteroplacental vascularity [24]. sFlt-1 can also quench vasodilatory factors, like PlGF, which are important for maternal vascular health. This ultimately leads to systemic reductions in nitric oxide (NO) bioavailability and endothelial dysfunction. Reduced NO has less capacity to activate its receptor, soluble guanylate cyclase (sGC), and production of the second messenger cyclic guanosine monophosphate (cGMP) resulting in maternal vascular dysfunction, hypertension, and intrauterine growth restriction (IUGR). In red font is the proposal that administration of NOS substrates or cofactors; modulators of sGC; or blocking the breakdown of cGMP with inhibitors of phosphodiesterase (PDE)-5 could be utilized to prevent the development, treat symptoms, or reduce the severity of PE.

NO produced by endothelial cells diffuses to surrounding platelets and the vascular smooth muscle cell (VSMC) layer where it binds to the heme moiety of soluble guanylate cyclase (sGC). sGC serves as the receptor for NO (Figure 1). sGC is an enzyme that catalyzes the conversion of guanosine triphosphate into cyclic guanosine monophosphate (cGMP). While cGMP inhibits platelet reactivity, it triggers the phosphorylation of multiple cell proteins and lower intracellular free calcium concentrations in VSMC, promoting vascular relaxation. Downstream in the cascade of NO/cGMP pathway are phosphodiesterases (PDE), a family of enzymes responsible for regulating the localization, duration, and amplitude of cyclic nucleotide signaling within the cell. PDE-5 is of particular importance for the degradation of cGMP in VSMC, thereby influencing vascular contractile tone [23].

## 3. NOS-Mediated Control of Renal and Systemic Vascular Function and Blood Pressure Regulation in Normal Pregnancy

There are dramatic hemodynamic changes that occur during normal pregnancy encompassing progressive vasodilation to allow for plasma volume expansion (PVE) and blood flow of nutrients to the growing uteroplacental–fetal unit [25]. This is accompanied by maintained or reduced maternal blood pressure by term [16]. NOS largely governs these physiological adaptations [26,27], whereby PVE is mediated by distinct local changes in renal NO signaling [28]. Urinary NOx, as a measure of renal NO production, progressively rises during gestation in normal pregnant rats [16]. NOS mediates the increased renal blood flow during pregnancy. This would typically promote sodium excretion but not during pregnancy because of attenuated renal tubular NO signaling due to increased PDE-5 activity to degrade the NO second messenger, cGMP [28]. Collectively, this attenuates the natriuretic effects of NO to allow for continued sodium reabsorption and PVE in the face of increased renal blood flow during normal pregnancy. This point has been demonstrated by studies using chronic administration of non-selective NOS inhibitors, like N(gamma)-nitro-L-arginine methyl ester (L-NAME). L-NAME dosing during early pregnancy attenuated the elevations in glomerular filtration (GFR), renal plasma flow (RPF), and PVE towards the end of pregnancy in rats [13,29].

**Figure 2:**
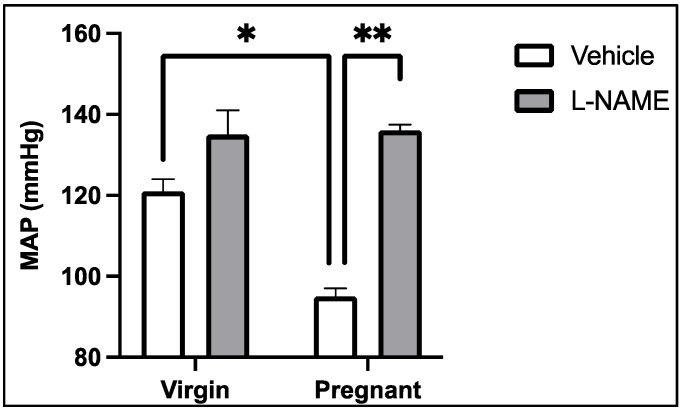
Impact of non-selective NOS inhibition with L-NAME on conscious maternal mean arterial blood pressure (MAP). L-NAME was administered in pregnant rats from gestational day 13–19 in drinking water (Vehicle). * *p* < 0.05 for virgin vs. pregnant vehicle-treated rats; ** *p* < 0.05 for pregnant + vehicle vs. pregnant + L-NAME rats. Mean ± SEM. Data adapted from [25].

L-NAME has multiple systemic effects during pregnancy. L-NAME administration in rats prevents the fall in systemic vascular resistance by mid-gestation [13]. At that time point, maternal blood pressure is not significantly different between non-pregnant, normal pregnant, or pregnant + L-NAME groups. However, it was found in a separate study that later administration of L-NAME from gestational days 13–19 produced a profound hypertensive response by the end of pregnancy. In this study, the pregnant rats had a greater degree of blood pressure elevation than did their non-pregnant counterparts [25] (Figure 2). Similarly, NOS inhibition from gestational days 7–20 promoted hypertension and reduced PVE in pregnant rats [29]. Recent data suggest that reduced PVE causes the uterine circulation to compensate for uteroplacental malperfusion by increases in NO-mediated vasodilation [30]. Nevertheless, this may not be sufficient to fully prevent the placental apoptosis that accompanies NOS inhibition during pregnancy [31], as the barotrauma of hypertension seems to feed forward to reduce uterine vascular blood flow in L-NAME-hypertensive pregnant rats [32].

The above studies utilized a pharmacological means to non-selectively inhibit the NOS enzymes, with agents such as L-NAME, during pregnancy. However, differential expression of the NOS isoforms has been reported during normal pregnancy. NOS3 protein expression progressively declines but NOS1 and NOS2 increase in the kidney during pregnancy in rats [16]. In order to define the specific role of individual NOS isoforms on maternal vascular function and blood pressure regulation during pregnancy, knockout-mouse experiments were required. Each NOS isoform has been knocked out and maternal and fetal outcomes examined to some extent. NOS3-knockout pregnant mice have reduced uterine blood flow toward the end of gestation [33] and elevated blood pressure measured via telemetry [34]. However, differing results have been reached using tail-cuff plethysmography with no difference or elevated systolic blood pressure compared to wild-type controls [35,36]. Results also indicate that NOS3-knockout mice had attenuated uterine artery diameter, uterine blood flow, and spiral artery remodeling along with signs of placental ischemia and reduced fetal weight [33]. It was found that NOS3 is an important signaling pathway whereby insulin-like growth factor 1 (IGF-1), which increases during normal pregnancy [37], promotes fetal growth [38].

In consideration of the other NOS isoforms, a study examined the impact of singly knocking out each and found no alteration in maternal blood pressure when measured by tail-cuff [35]. Moreover, the number of viable offspring was not altered by single-knockout of the individual NOS isoforms; but double-knockout of any two NOS genes reduced offspring number to a similar extent; and the triple-knockouts had further reductions in this pregnancy outcome [39]. Unfortunately, pregnancy was not a major focus of that study, and thus, a more in-depth examination of placentation, trophoblast viability, uteroplacental vascular outcomes, fetal growth restriction, and maternal blood pressure were not examined following the different permutations of knocking out the NOS isoforms.

The earlier genetic strategies manipulating the expression of each NOS isoform have a caveat due to the fact that there are splice variants of NOS1, including NOS1-α, -β, and -γ [40]. The older NOS1 knockout studies only targeted NOS1-α expression [41]. Recent research has led to the development of NOS1-β knockouts that revealed important mechanisms for intrarenal control of systemic blood pressure during pregnancy [42]. As mentioned, normal pregnancy is accompanied by tremendous vasodilation noted by increases in GFR and RPF. Elevations in GFR during normal pregnancy are mediated by attenuated tubuloglomerular feedback (TGF). This allows for glomerular hyperfiltration even though there is lower chloride, which is owed to PVE, delivered to salt-sensing cells in the distal nephron, called the macula densa [43,44]. This would typically activate a negative-feedback loop to stabilize the tubular flow reaching the macula densa and allow for proper sodium excretion. This process is mediated via increased NOS1-β signaling in the macula densa, whereby in the non-pregnant state, macula densa-specific NOS1-β-knockout mice have enhanced TGF and fail to increase GFR in response to acute volume expansion producing a salt-sensitive hypertension phenotype [45]. During normal pregnancy, expression of NOS1-β in the macula densa increases in rodents and humans, and its knockout leads to enhanced TGF, decreased GFR, and causes hypertension to mimic the blood pressure phenotype in preeclamptic women [42].

It is not yet fully known what drives the increase in macula densa NOS1 during normal pregnancy, but pregnancy-specific rises in hormones could be responsible. This includes relaxin, which is produced from the ovaries and placenta. Relaxin administration increased renal NO metabolite excretion, but this was examined only in male rats [46]. Relaxin is reduced in preeclamptic women [47]. The impact of relaxin on blood pressure regulation has been examined in a preclinical model of PE, namely the Reduced Uterine Perfusion Pressure (RUPP) rat model. This model is produced by strategically placing silver clips around blood vessels within the pregnant uterus, eliciting uteroplacental malperfusion and placental ischemia-induced hypertension. RUPP rats have many similarities to women with PE [48]. Administration of a recombinant form of human relaxin-2, named serelaxin, increased plasma NOx and attenuated the development of hypertension in RUPP rats [49]. However, it has not yet been examined how serelaxin impacts renal NOS expression or NOx excretion, especially as RUPP rats have a downregulation of NOS1-β in the macula densa [42]. As NOx is reduced in PE, the next section will explore evidence to support that maternal NO bioavailability is attenuated by soluble placental ischemic factors to promote hypertension in PE.

## 4. Soluble Placental Ischemic Factors and Reduced NO in PE

Studies conducted since the 1990s have shown that plasma and 24 h urinary NOx or cGMP are reduced or do not rise in women with PE compared to normal pregnancy [50,51]. These metabolites are important to measure because they not only provide an indication of NO bioavailability, but nitrite has a biological activity to promote vasorelaxation in human placental vessels [52]. Within the past 5 years, the majority of studies have shown that preeclamptic women and experimental animal models have reduced measures of circulating NO bioavailability, like NOx, and lower levels of NOS3 (Table 1). Moreover, in vivo techniques to assess vascular function have demonstrated that women with PE have vascular dysfunction [53,54] and increased vascular resistance [55]. This vascular dysfunction is associated with circulating levels of placental ischemic, hypertensive factors, such as increased serum soluble fms-like tyrosine kinase (sFlt-1). Elevated sFlt-1 quenches the vasodilatory capacity of vascular endothelial growth factor (VEGF) and placental growth factor (PlGF). NOS largely contributes to this dilatory response in uterine arteries isolated from late-pregnant rats [56]. An elevated sFlt-1:PlGF ratio is a biomarker for those women that are more at risk for PE with severe features [54,57].

In the experimental animal setting, models generated by reducing uterine perfusion or by producing chronic excess of exogenous placental ischemic factors, like sFlt-1, have been used to probe the severe features of PE. Experimental animal models ranging from nonhuman primates to rodents confirm that reductions in uteroplacental blood flow elicits placental ischemia/hypoxia-induced release of soluble factors that promote endothelial and vascular dysfunction and hypertension in PE [48,85,106,107]. Indeed, reducing sFlt-1 with siRNA technology attenuated uteroplacental ischemia-induced hypertension and proteinuria in pregnant baboons [108]. That study did not examine maternal NO bioavailability, but this has been touched upon in rodent models of PE. The infusion of sFlt-1 into once normotensive pregnant rats produced hypertension and reduced glomerular generation of NO, as measured by DAF fluorescence [109]. The precise mechanisms whereby this occurs are not fully clear, but sFlt-1 infusion into pregnant mice reduced systemic vascular mRNA expression of endothelin type B (ET_B_) receptors [110]. This receptor serves to mediate the vasodilatory action of the endothelium-derived vasoactive peptide, endothelin-1 (ET-1), by stimulating NOS enzymatic activity [111]. It has begun to be examined if reductions in ET_B_ potentiate placental ischemia-induced hypertension. We have data to support that ET_B_-deficient rats have increased blood pressure by the end of pregnancy and this response is exaggerated in the face of the RUPP procedure [112].

**Figure 3:**
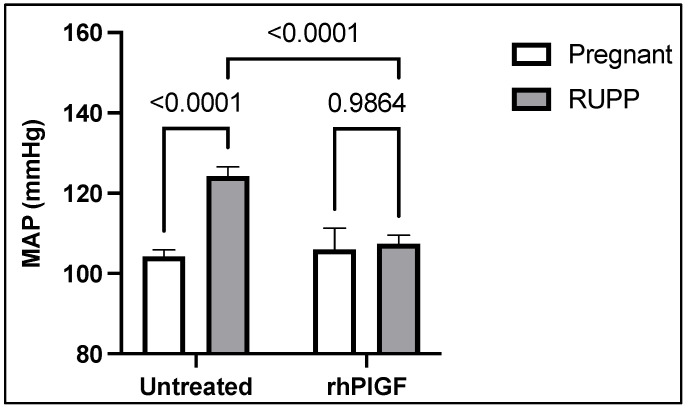
Effects of treatment with recombinant human placental growth factor (rhPlGF) on blood pressure responses in pregnant rats with reduced uterine perfusion pressure (RUPP). Rats were subjected to the RUPP procedure on gestational day 14. Rats were administered rhPlGF (180 μg/kg per day, I.P. osmotic minipump) from gestational day 14–19. Conscious mean arterial blood pressure (MAP) was measured on day 19. *p*-Values appear in the above brackets. Mean ± SEM. Data adapted from [116].

Telemetric evidence supports the development of placental ischemia-induced hypertension in RUPP rats [113], which is accompanied by reduced NOS-mediated buffering of vascular tone and increased vasoconstriction [114]. Furthermore, we have shown that RUPP-hypertensive rats have elevated circulating sFlt-1 and administration of recombinant human PlGF prevented the development of their hypertension (Figure 3). This has been supported by others [94]. However, it has not been examined whether this anti-hypertensive effect of PlGF is mediated by NOS, which could be studied by dosing with L-NAME. It could be that RUPP rats with complete NOS inhibition with L-NAME would present with more severe features of PE. Moreover, it is currently unknown whether a deficiency in any specific NOS isoform impacts the response to placental ischemia-induced hypertension. What is known is that NOS3-knockout mice have exaggerated hepatic dysfunction, thrombocytopenia, renal injury, and hypertension in response elevated sFlt-1 induced by adenoviral overexpression [115]. Together, these studies support that NOS deficiency exaggerates placental ischemia-induced hypertension, but far less is understood about whether this pathway is targeted to increase the risk for PE in the face of emerging cardiovascular disease risk factors.

## 5. Pro-Inflammatory States as Risk Factors for Placental Ischemic Disease and PE

A major risk factor for PE is obesity [117]. Obesity is known as a pro-inflammatory state [118]. Women with combined obesity and PE have greater blood pressure levels and markers of increased inflammation measured by members of the tumor necrosis factor (TNF) family of proteins [119]. Adverse diets are a cause of obesity. A pro-inflammatory diet, in addition to perceived psychological stress, is associated with greater circulating TNF-α in pregnant women [120]. High-fat diet feeding results in elevated TNF-α levels and expression of the pro-inflammatory NOS isoform, NOS2, in placentas from pregnant mice [121,122,123]. There is conflicting data about NOS2 expression in placentas isolated from obese women [124,125]. However, maternal blood pressure is not always a focus of such studies. It has been shown that administering the NOS2 inhibitor, 1400 W, prevented RUPP-induced hypertension in rats [126]. NOS2 has been found to be increased in women with PE (Table 1).

Another inflammatory pathway studied in PE is the complement system. This system is important to “complement” the ability of antibodies and phagocytic cells to effectively remove microbes and apoptotic debris before they are able to release pro-inflammatory molecules. There are numerous small proteins involved in the complement signaling cascade. Notably, one such protein is C1q, which is expressed by trophoblast cells [127]. Deficiency of C1q in mice resulted in attenuated placental development and vascular remodeling. Further evidence that C1q expressed by trophoblast cells is important was demonstrated by the finding that paternal deficiency alone, and not the maternal deficiency of C1q, resulted in a PE-like phenotype in wild-type female mice presenting with increased blood pressure, increased fetal demise, and systemic vascular dysfunction during late-pregnancy [128,129]. Here, circulating sFlt-1 nor PlGF levels were altered at mid- or late-pregnancy. However, it was found that sFlt-1 levels in serum were higher when both male and female breeders were deficient in C1q [129]. Circulating levels of C1q are lower in women with PE [130]. C1q is involved in the classical pathway of complement function, but overactivation of proteins associated with alternative, pro-inflammatory complement signaling, including C3a, have been shown to mediate the development of placental ischemia-induced hypertension in the RUPP rat [131].

Dysfunctional signaling within the complement cascade is associated with loss of self-tolerance and autoimmunity [132]. The autoimmune disease, systemic lupus erythematosus (SLE), has a propensity to affect women of reproductive age and increases the chance of complications during pregnancy, like PE [133]. It has been found that there is an increased sFlt-1:PlGF ratio during early pregnancy in women with SLE [134], which is indicative of placental ischemic disease in the face of maternal inflammation. 

There is more support that maternal infection increases the incidence of placental ischemia and the onset of severe PE. One type of infection that is currently very prominent is exposure to the SARS-CoV-2 coronavirus, the cause for COVID-19. It has been linked to a greater risk for severe PE [135,136,137,138]. It was recently reviewed that almost 38% of pregnancies infected by COVID-19 have markers of uteroplacental malperfusion, including fibrin deposition, infarction, decidual vasculopathy, accelerated villous hyperplasia, distal villous hypoplasia, and retroplacental hemorrhage, as well as placental inflammation [139,140].

Placental malperfusion with ischemia/hypoxia not only elicits placental and maternal pro-inflammatory factors like TNF-α and agonistic autoantibodies to vasoconstrictor systems, like the angiotensin II type 1 receptor (AT1-AA) and α-adrenergic receptor, but also results in significant increases in placental and plasma levels of sFlt-1 [5]. TNF-α infusion increases blood pressure and circulating levels of sFlt-1 and AT1-AA in rats [141]. However, lacking is an optimal way to intervene in the signaling of these pro-inflammatory factors. These factors are consistently associated with reduced maternal NO bioavailability. It has not been thoroughly explored whether strategies to increase NOS coupling attenuates the development of PE.

## 6. Potential Treatment Strategies Targeting to Increase NO Bioavailability in PE

Treatment strategies have been tested in the settings of PE, attempting to increase NO bioavailability. In this section, we briefly summarize studies in humans and experimental animals evaluating potential therapies targeting the NOS system for ameliorating placental and/or vascular dysfunction in PE, with a focus on supplementation of NOS substrates and/or cofactors, modulators of sGC, and inhibitors of PDE-5. The role of NO donors, including organic nitrates and S-nitrosothiols, to attempt to prevent and ameliorate the clinical manifestation of PE has been extensively reviewed elsewhere [142,143].

### 6.1. L-Arginine Supplementation

L-arginine levels determined by chromatographic methods have been found to be reduced in maternal and umbilical cord plasma of preeclamptic women [144,145,146,147]. When assessed in the first trimester of gestation, plasma L-arginine levels were decreased in those women developing early-onset PE [148]. Promisingly, clinical studies demonstrated that intravenous and/or oral treatment with L-arginine improves many features of PE, such as hypertension, pre-term birth, and low birth weight [149,150,151,152]. In addition, L-arginine supplementation initiated anytime from 14 to 32 weeks of gestation significantly prevented the development of PE in patients deemed at risk for this syndrome [153,154]. However, few placebo-controlled trials reported no beneficial effects of L-arginine supplementation in PE [155,156], possibly due to differences in treatment initiation, dosage, and duration. Nonetheless, meta-analyses including these and additional studies concluded that L-arginine is superior to placebo in lowering blood pressure and prolonging pregnancy in women with established PE as well as in reducing the incidence of PE in high-risk women [157,158]. Studies in experimental animals also support the human data showing that treatment with L-arginine ameliorates hypertension during pregnancy [109,159,160,161]. For instance, 2% L-arginine added to the drinking water of RUPP rats or sFlt-1-infused pregnant rats significantly decreased their blood pressure levels, likely by increasing NO bioavailability and downregulating renal endothelin-1 expression [109,159]. These studies provide evidence that L-arginine supplementation during pregnancy is safe and may be used as a preventive and/or therapeutic tool in PE. As such, larger randomized double-blinded trials examining L-arginine supplementation in PE should be encouraged.

### 6.2. BH4 Supplementation

Scarce studies have assessed BH4 in PE. Kukor et al. found that, although BH4 levels were similar in placental tissue of PE and normal pregnant women, placental NOS3 activity exhibited two distinct responses to BH4 in PE: in placental homogenates from few PE patients (n = 3), the addition of physiological and higher concentrations of BH4 stimulated NOS3 activity similar to that of normal placental homogenates, whereas for the majority of PE placental homogenates (n = 7), only the addition of supraphysiological concentrations of BH4 caused significant NOS3 stimulation [162]. Using an animal model PE induced by injecting deoxycorticosterone acetate once a week and adding 0.9% saline to the drinking water (DOCA-salt) of female Sprague–Dawley rats before mating and during pregnancy, Mitchell et al. showed that ex vivo treatment with sepiapterin, a BH4 precursor, normalized decreased endothelium-dependent relaxation responses of mesenteric arteries, reduced NO, and increased superoxide and peroxynitrite levels of aortic tissue [163]. Similarly, incubation of mesenteric arteries with sepiapterin restored the decreased endothelium-dependent vasodilation of pregnant mice with deficiency of a copy of the *cystathionine-beta synthase* gene [164]. These heterozygous mice develop moderate hyperhomocysteinemia, a condition associated with PE and later cardiovascular disease [165,166,167]. Studies evaluating the in vivo effects of BH4 supplementation in PE have yet to be conducted in humans and experimental animals. However, clinical studies revealed that acute infusion of BH4 improved the impaired endothelium-dependent vasodilation in hypertensive patients to the level of normotensive counterparts [168]. Moreover, chronic oral treatment with 5 or 10 mg/kg/day of BH4 for 8 weeks, or 400 mg of BH4 in divided doses for 4 weeks, ameliorated endothelial function and blood pressure in human subjects with poorly controlled hypertension [169]. Studies in different animal models of chronic hypertension, such as those in spontaneously hypertensive rats, 5/6 nephrectomies rats, and angiotensin II-infused rats, reinforce that BH4 supplementation is able to improve hemodynamics and NO/cGMP signaling [170,171,172,173,174,175]. But, those studies did not focus on pregnancy hypertension. Overall, these findings suggest that BH4 supplementation deserves further consideration as a potential therapy for PE.

### 6.3. L-Citrulline Supplementation

Maternal and umbilical cord serum levels of L-citrulline have been reported to be similar in PE and normal pregnancy [149,176]. However, there is evidence arguing that circulating L-citrulline levels are reduced in women prone to develop recurrent PE [177] but elevated in women presenting severe PE [178]. Notably, L-citrulline content is decreased in human umbilical vein endothelial cells (HUVECs) isolated from late-onset preeclamptic women [75]. Nonetheless, L-citrulline instead of L-arginine has been proposed as a better supplementation strategy with regards to blood pressure and fetal growth because it bypasses hepatic first-pass metabolism and is converted to L-arginine within tissues [179,180]. L-citrulline supplementation has been tested in pregnant mice with deficiency of a copy of the complement component *C1q* gene, an animal model that exhibits pregnancy-specific vascular dysfunction, hypertension, proteinuria, and impaired fetal growth. The addition of 0.25% L-citrulline to the drinking water of these animals throughout gestation improved blood pressure, endothelium-dependent and -independent relaxation of mesenteric arteries, fetal weight, and placental efficiency [181]. L-citrulline supplementation in drinking water (2 g/kg/day, from gestational day 7 to 21) also increased fetal weight in a rat model of intrauterine growth restriction (IUGR) induced by maternal dietary protein restriction, probably via enhanced NO production and expression of genes related to placental angiogenesis and survival [182,183]. Despite these preclinical studies showing that L-citrulline supplementation improves pregnancy outcomes, there are no human studies to date examining the impact of L-citrulline treatment on PE. Pooling data from randomized clinical trials, a recent meta-analysis performed by Barkhidarian et al. concluded that L-citrulline, when supplemented at a dose ≥6 g/day, decreases both systolic and diastolic blood pressures in non-pregnant subjects [184]. Thus, future clinical studies should evaluate the effects of L-citrulline supplementation in PE.

### 6.4. Downstream Targets: sGC Stimulators, sGC Activators, and PDE-5 Inhibitors

Although the aforementioned studies investigating L-arginine, BH4, or L-citrulline as a therapeutic intervention in PE are promising, drugs targeting downstream mechanisms in the NO/cGMP pathway might be a better option for the treatment of preeclamptic women carrying functional alterations in the *NOS3* gene. Several clinical studies associated *NOS3* polymorphisms with increased risk of PE, which has been summarized by two recent meta-analyses confirming that the presence of the polymorphic allele at the 894 (T instead of G) position in the *NOS* gene predisposes pregnant women, especially those with Caucasian background, for the development of PE [185,186]. It has been previously shown that the G894T polymorphism affects NOS3 activity and cellular localization, leading to decreased NO formation in carriers of the T allele [187,188]. Indeed, the T allele for the G894T polymorphism has been associated with reduced circulating NO levels in both normal pregnancy and PE [189,190]. Additional studies have found that other commonly associated *NOS3* polymorphisms with PE may also alter circulating NO levels [191,192]. Thus, in those situations where NOS activity is compromised by genetically driven defects, supplementation with substrates and/or cofactors might not act as expected, and alternative strategies should be explored.

Data regarding maternal blood/urine levels of cGMP in PE have been variable with studies describing reduced [51,61,193,194], elevated [195,196,197,198,199], or statistically unchanged levels [50,200,201,202,203]. Reduced cGMP levels are likely due to impaired NOS3 activity and decreased NO levels [51,61,194], whereas elevated cGMP levels may result from increased activation of sGC by atrial and/or brain natriuretic peptides in PE [196,197,198,199]. Nevertheless, it seems that sGC expression and activity are decreased in decidual and placental tissues collected from preeclamptic patients [204,205]. Studies in models of PE, including RUPP rats, pregnant rats administered the sulfonic acid, suramin, and the Dahl salt-sensitive rat model of superimposed PE corroborate findings in humans indicating that sGC expression, as well as cGMP levels, are reduced in blood vessels [206,207,208]. sGC stimulators and activators are a novel class of drugs that modulate sGC to increase cGMP production independently of NO. While sGC stimulators such as riociguat bind directly to the reduced, heme-containing form of the enzyme, sGC activators like cinaciquat bind to its oxidized, heme-free form [209]. Treatment of RUPP rats with an sGC activator added to the diet (BAY 60–2770, 16 ppm, ad libitum) from gestational day 14 to 19 restored their reduced cGMP levels and endothelial function of uterine arteries, reflecting on the amelioration of blood pressure [210]. Similar results were found along with improved uteroplacental blood flow, placental remodeling, and fetal growth were obtained by treating RUPP rats with daily subcutaneous injections of a sCG stimulator (riociguat, 10 mg/kg/day) from gestational day 14 to 20. However, sham pregnant rats undergoing the same therapy regimen with riociguat exhibited impaired uteroplacental blood flow and placental remodeling similar to vehicle-treated RUPP rats [206]. Follow-up studies revealed that, despite the effect of riociguat on prolonging pregnancy of RUPP rats, it worsened the probability of their babies surviving at birth and postnatal day 2. Moreover, riociguat treatment during late pregnancy did not mitigate RUPP-induced asymmetric IUGR and increased cardiovascular risk in male offspring at 4 months of age [211]. Importantly, although the US Food and Drug Administration (FDA) agency has approved riociguat (Adempas, Bayer) for the treatment of pulmonary arterial hypertension (PAH) and chronic thromboembolic pulmonary hypertension (CTEPH), it is highlighted in its prescribing information that this medication has embryo-fetal toxicity and should not be administered to pregnant women. Thus, further studies in preclinical models of PE should be carried out to directly compare the maternal and fetal outcomes of sGC activators, stimulators, and PDE-5 inhibitors.

Reduced levels of cGMP in PE may also be due to increased PDE-5 activity. Indeed, clinical studies evaluating this cGMP-degrading enzyme in PE reported enhanced circulating PDE activity [212], with data in the rat RUPP model demonstrating increased PDE-5 expression in renal medullary and placental tissue [213]. Sildenafil and tadalafil have been tested clinically as PDE-5 inhibitors for the treatment of adverse pregnancy outcomes in PE. Earlier randomized controlled trials with sildenafil were promising, indicating beneficial effects on blood pressure, UARI, and duration of pregnancy in PE, with no increase in maternal or fetal morbidity and mortality [214,215,216]. In contrast, the Sildenafil Therapy in Dismal Prognosis Early Onset Fetal Growth Restriction (STRIDER) trial was prematurely terminated due to concerns that sildenafil may cause neonatal pulmonary hypertension, whereas benefit on perinatal mortality or major neonatal morbidity was unlikely [217,218]. Ferreira and collaborators’ meta-analysis evaluating sildenafil for the prevention or treatment of obstetric diseases concluded that it increases fetal weight at birth in the settings of placental insufficiency; however, they queried randomized clinical trials published up to September 2018, thereby not considering the results from the STRIDER trial [219]. Furthermore, a multicenter phase II clinical trial concluded that tadalafil, although safe, did not prolong pregnancy duration in PE [220]. A subsequent clinical study with preeclamptic patients treated with tadalafil found a dose-dependent increase in maternal mild adverse events (headache and palpitation), but all administered dosages were deemed safe for both mother and fetus [221]. Hence, a new meta-analysis should be performed, including the results of these recently published clinical trials on PDE-5 inhibitors in PE. 

Numerous preclinical studies in PE have been conducted with sildenafil and tadalafil. Yet, most of these studies have utilized the mouse or rat L-NAME model. Although we agree that this animal model is valid and reiterates the importance of NOS on regulating placentation, vascular function, and blood pressure during pregnancy, it was already expected that the treatment of these animals with drugs targeting the same pathway being disturbed would lead to successful maternal and fetal outcomes [222,223,224,225,226,227]. Nonetheless, studies with *catechol-O-methyl transferase* knockout pregnant mice, RUPP rats, Dahl-salt sensitive rat model of PE agree with the findings in the mouse/rat L-NAME model, showing that sildenafil improves endothelial function, blood pressure, UARI, and fetal growth in PE [213,228,229]. Therefore, the use of PDE-5 inhibitors in PE is controversial and future studies should distinguish their effects between early- versus late-onset PE.

## 7. Summary and Conclusions

The maternal vascular endothelium appears to be an important target for factors involved in the pathophysiology of PE. The endothelium normally controls the balance between competing factors that ultimately impact vascular tone, coagulation, platelet function, and fibrinolysis. One endothelial factor that appears to play an important role in PE is NO. Not only does NO play an important role in the regulation of renal function and arterial pressure under various physiological and pathophysiological conditions, growing evidence suggests that reduced NO synthesis plays a central role in the pathophysiology of PE. In normal pregnancy, increased NO mediates renal vasodilation and decreases total peripheral resistance and blood pressure. However, in women with PE and in various animal models of PE, NO production is reduced, resulting in attenuated endothelium-dependent dilation, and the vasculature is hyper-responsive to a myriad of vasoconstrictive stimuli as a result of placental dysfunction. Some of these factors include sFlt-1, soluble endoglin, AT1-AA, and inflammatory cytokines.

Although there has been progress in understanding the mechanisms responsible for the pathogenesis of PE, effective therapeutic options for women that develop PE are still not available. This review highlights the concept that agents that improve NOS coupling and signaling through sGC to directly target the endothelial dysfunction could serve as potential therapies to alleviate the maternal symptoms of PE to prolong pregnancy in severe PE (Figure 1). While preclinical studies in a number of animal models for studying PE have demonstrated beneficial effects of agents that impact NOS signaling, further investigation of the efficacy and safety of these agents is greatly needed. 

## Figures and Tables

**Table 1:** PubMed search results for keywords “nitric oxide” AND “preeclampsia” form 2015-pr. Arrows represent the direction of change (↑, increased; ↓, decreased), and equal signs (=) represent no change in NOS expression/NO biomarkers (NOx).

Species/Experimental Model	Circulating	Tissue
Human	Shaheen G. et al. [58] ↓ NOx Possomato-Vieira J.S. et al. [59] ↓ NOx Pereira D.A. et al. [60] ↓ NOx McCann Haworth S.M. et al. [61] ↓ NOx Tashie W. et al. [62] ↓ NOx Kim S. et al. [63] ↑ NOx Mazloomi S. et al. [64] ↓ NOS Lai H. et al. [65] ↓ NOx Ajadi I. et al. [66] ↓ NOx Serrano-Berrones et al. [67] ↓ NOx Deniz R. et al. [68] ↓ NOx Bos M. et al. [69] ↓ NOx ElMonier A.A. et al. [70] ↓ NOx Hodzic J. et al. [71] ↓ NOx Rocha-Penha L. et al. [72] ↓ NOx Bambrana V. et al. [15] ↓ NOx Lai H. et al. [65] ↓ NOx	Blood vessels: Lorca R.A. et al. [73] ↓ NOS function Primary HUVECs: Chen J. et al. [74] ↓ NOS3 Salsoso R. et al. [75] ↓ NOS activity Placenta: Mishra J.S. et al. [76] ↓ NOS3 K.-Y. Jung et al. [77] ↑ NOS2 Kim S. et al. [63] ↑ NOS2, ↓ NOS3 Mukosera G.T. et al. [78] ↑ NOx Shaheen G. et al. [79] ↓ NOS3 Guerby P. et al. [80] ↓ NOS3
		Hitzerd E. et al. [81] ↑ maternal placenta NOS3, ↓ maternal placental NOS2, = fetal placenta NOS2, ↑ fetal placenta NOS2 Guerby P. et al. [82] ↓ NOS3 Li F.F. et al. [55] ↓ NOS2, NOS3 Motta-Mejia C. et al. [83] ↓ NOS3 Du L. et al. [84] ↑ NOS2, ↓ NOS3
Non-human primate/EarlyPregnancy Excess of Estradiol	Albrecht E.D. et al. [85] ↓ NOx	Blood vessels: Albrecht E.D. et al. [85] ↓ NOS3
Rat/RUPP	Travis O.K. et al. [86] ↓ NOx Palei A.C. et al. [17] ↓ NOx Cottrell J.N. et al. [87] ↓ NOx El-Saka M.H. et al. [88] ↓ NOx Wang C. et al. [89] ↓ NOx Amaral L.M. et al. [90] ↓ NOx Jammalamadaga V.S. et al. [91] ↓ NOx Santiago-Font J.A. et al. [49] ↓ NOx	Blood vessels: Younes S.T. et al. [92] = NOS3 Ma S.L. et al. [93] ↓ NOS3, NOx Zhu M. et al. [94] ↓ NOS3 Placenta: Tengfei Z. et al. [95] ↓ NOS3, ↑ NOS2 Wang C. et al. [89] ↓ NOS3
Rat/DOCA-salt	Wang G.-J. et al. [96] ↓ NOx	Placenta: Chimini J.S. et al. [97] ↓ NOx Tyurenkov I.N. et al. [74] ↓ NOS3, ↑ NOS2
Rat/Elevated Testosterone	Mishra J.S. et al. [76] ↓ NOx	Blood vessels: Mishra JS et al. [76] ↓ NOS3
Rat/Lipopolysaccharide (LPS)	Ou M. et al. [98] ↓ NOx Hu J. et al. [99] ↑ NOx	-
Mouse/AntiphospholipidSyndrome	Lefkou E. et al. [100] ↓ NOx	-
Mouse/Human PE Serum Injection	Purnamayanti N.M.D. et al. [101] ↓ NOx	-
Mouse/Prolactin Overexpression	-	Kidney: Chang A.S. et al. [102] ↓ NOx, ↑ NOS2
Mouse/Progranulin Deficiency	-	Placenta: Xu B. et al. [103] ↓ NOS3
Mouse/Hypoxia Chamber	-	Blood vessels: Lane S.L. et al. [104] ↓ NOS function
Mouse/sFlt-1 Adenovirus	-	Blood vessels: Zhang S. et al. [105] ↓ NOS3
